# Supplementary material for: Saccharomyces boulardii (CNCM I-745) improves intestinal damage in sepsis by remodeling intestinal flora structure
Source: Front Cell Infect Microbiol. 2025 Jul 31;15:1602792. doi: 10.3389/fcimb.2025.1602792 (PMC12350329; doi:10.3389/fcimb.2025.1602792)
Supplement: Supplementary file 1 [file DataSheet1.zip › summary/summary/6_Taxonomy_community/2_Bubble_plot/bubble_plot_Group.pdf]

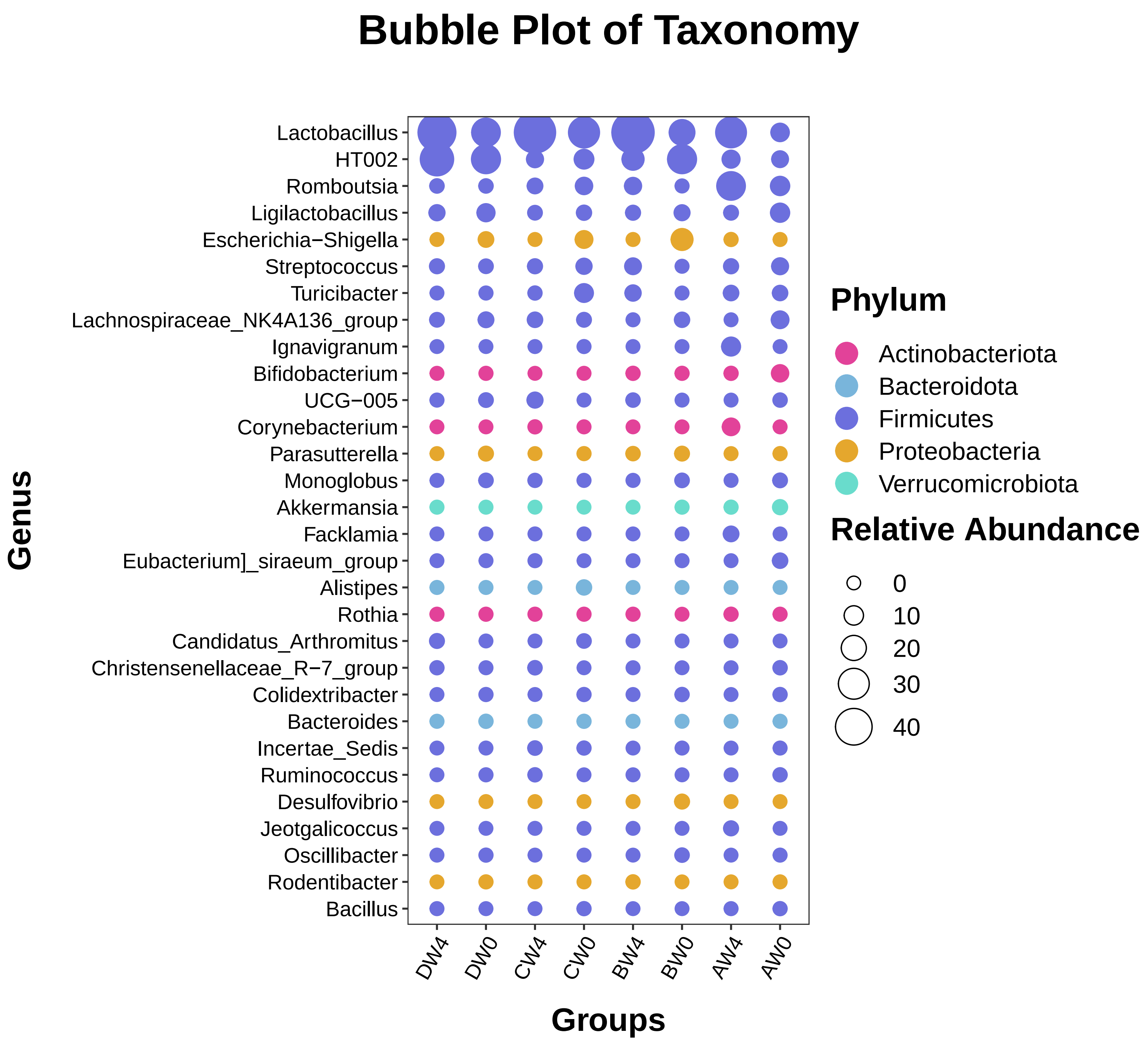
Bubble Plot of Taxonomy
Genus
Lactobacillus
HT002
Romboutsia
Ligilactobacillus
Escherichia−Shigella
Streptococcus
Turicibacter
Lachnospiraceae_NK4A136_group
Ignavigranum
Bifidobacterium
UCG−005
Corynebacterium
Parasutterella
Monoglobus
Akkermansia
Facklamia
Eubacterium]_siraeum_group
Alistipes
Rothia
Candidatus_Arthromitus
Christensenellaceae_R−7_group
Colidextribacter
Bacteroides
Incertae_Sedis
Ruminococcus
Desulfovibrio
Jeotgalicoccus
Oscillibacter
Rodentibacter
Bacillus
DW4
DW0
CW4
CW0
BW4
BW0
AW4
AW0
Groups
Phylum
Actinobacteriota
Bacteroidota
Firmicutes
Proteobacteria
Verrucomicrobiota
Relative Abundance
0
10
20
30
40
